# Cellular Imaging of Cadmium in Resin Sections of Arbuscular Mycorrhizas Using Synchrotron Micro X-ray Fluorescence

**DOI:** 10.1264/jsme2.ME13093

**Published:** 2013-02-06

**Authors:** Keiichiro Nayuki, Baodong Chen, Ryo Ohtomo, Yukari Kuga

**Affiliations:** 1 Graduate School of Integrated Arts and Sciences, Hiroshima University, 1–7–1 Kagamiyama, Higashihiroshima, Hiroshima, 739–8521 Japan; 2 Graduate School of Agriculture, Shinshu University, 8304 Minami-Minowa, Kamiina, Nagano 399–4598, Japan; 3 Nasu Research Station, NARO Institute of Livestock and Grassland Science, 768 Senbonmatsu, Nasushiobara, Tochigi, 329–2793 Japan

**Keywords:** arbuscular mycorrhiza, high-pressure freezing technique, synchrotron micro XRF, polyphosphate, EDS-SEM

## Abstract

Arbuscular mycorrhizal (AM) fungi function as extended roots and take an active part in plant acquisition of nutrients and also soil pollutants, such as heavy metals. The objective of this study was to establish a method to observe the localization of cadmium (Cd) Kα at subcellular levels using X-ray fluorescence (XRF) imaging with a synchrotron irradiation microbeam in resin-embedded sections of mycorrhizas. To evaluate the methodology, distributions of Cd in high-pressure-frozen *Lotus japonicus*—*Rhizophagus irregularis* mycorrhizal roots were compared between two treatments; Cd was exposed either to the roots or to the extraradical hyphae. Results showed that, in the latter treatment, Cd was restricted to fungal structures, whereas in the former, Cd was detected in cell walls of the two organisms. Plunge-frozen extraradical mycelium of *Gigaspora margarita* exposed to Cd showed high signals of Cd in the cell walls and vacuoles, and low in the cytoplasm. With selective staining and elemental mapping by electron-dispersive X-ray spectrometry (EDS), a positive correlation between distributions of Cd and P was revealed in the vacuole, which suggested polyP as a counter ion of Cd. These results indicated that there was no Cd relocation in rapidly frozen resin-embedded materials, therefore supporting the usefulness of this methodology.

Arbuscular mycorrhizal (AM) associations formed between plant roots and soil fungi are the oldest form of plant symbiosis recorded. AMs are categorized as endomycorrhiza and considered to be ancestral among 7 recognized mycorrhizal types, found in all major groups of extant plants (6). Fungi forming mycorrhizas are composed of two parts, intra- and extraradical hyphae, and essentially serve as extensions of roots; extraradical hyphae absorb mineral nutrients, such as phosphate (P) and nitrogen (N), from soil and translocate them to intraradical hyphae, which are then transferred to the host. The fungi, in return, receive carbon compounds from the host plants via intraradical hyphae, and then translocate them to extraradical hyphae and developing spores.

Cadmium (Cd) is a trace element in the environment. Human activities, such as the production of batteries, mining, and usage of rock phosphate fertilizer, have caused Cd contamination in soils (1, 17). Cd is not known as a biologically essential element and is toxic at higher concentration. Organisms accumulate Cd, to some extent, by absorbing from the surrounding environment and through food-webs; therefore, Cd pollution in soils is a food-safety issue in crop production (14). In contrast, plants that accumulate higher concentrations of Cd have attracted considerable attention for decontamination from polluted soils (15). Mycorrhizal plants often show higher Cd tolerance than non-inoculated control plants, and it has been reported that mycorrhizal colonization leads to an increase in the accumulation of Cd in maize roots but a decrease in the shoots (4, 10).

To determine how mycorrhizal fungi function in Cd accumulation in roots, a histological approach is essential to analyze elemental flow, because host and fungal cells are integrated into endosymbiotic tissues. For elemental mapping at the organelle/cellular level, EDS combined with TEM, EDS-SEM, and energy-filtering TEM (EF-TEM) have been commonly used. However, for Cd in biological materials, EDS is not suitable for detection; energy peak of characteristic X-ray fluorescence (XRF) of Cd overlaps with that of potassium, a common element by mass in cells. Highenergy synchrotron radiation is a powerful tool to detect Cd by its K line XRF (23.11 keV), which eliminates the possibility of signal contamination from co-existing lighter elements.

The objective of this study was therefore to establish a method to localize Cd at cellular levels, excluding interference from the signals of other cellular elements, such as potassium, on mycorrhizal fungi composed of extraradical hyphae colonizing in soil and intraradical hyphae together with plant tissue. For this purpose, samples of both extraradical hyphae and mycorrhizal roots were prepared for resin embedding by either immersion of tissues in liquid propane or high-pressure freezing, followed by freeze substitution. On thick resin-embedded sections, the microscale distributions of Cd and Zn were determined by synchrotron microbeam XRF (SR-μXRF) by detecting fluorescence intensities of Cd Kα and Zn Kα. This paper focuses on technical issues, such as the strategy and evaluation of the method based on results obtained from different Cd treatments and from cellular distribution of Cd.

## Materials and Methods

### Arbuscular mycorrhizal plant settings

**Fig. 1:**
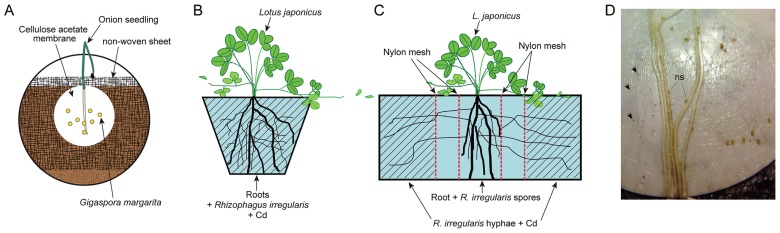
Inoculation and Cd treatment systems of *Allium cepa* and *Gigaspora margarita* (A & D), and *Lotus japonicus*—*Rhizophagus irregularis* (B & C) arbuscular mycorrhizas. (A) A plastic Petri dish root box system (90 mm diam, 15 mm depth.). From 80 to 86 dai, 50 ppm Cd nitrate in water was added every day until harvest. (B) One-compartment system (C1). A seedling and spores were cultivated in Cd-contaminated soil (200 ppm Cd chloride). (C) Five-compartment system (C5). An acrylic box (14 cm length × 10 cm width × 10 cm depth) was divided into 5 by nylon mesh (37 μm), which only hyphae could pass through. The middle three compartments contained only soil (field soil : river sand = 2:1) and the two outermost compartments contained Cd-contaminated soil (200 ppm Cd chloride, field soil : river sand = 3:1). Seedlings and spores were placed in the center compartment, which was sandwiched between two buffer compartments; therefore, only extraradical hyphae of *R. irregularis* extended into the outermost compartments and were exposed to Cd. (D) An extraradical hyphal network on a cellulose acetate membrane by establishment of *A. cepa*—*G. margarita* arbuscular mycorrhiza. Arrows, auxiliary cells; ns, newly formed spore.

*Allium cepa* (onion)—*Gigaspora margarita* Becker & Hall (MAFF520054, Ministry of Agriculture, Forestry and Fisheries Genebank, Tsukuba, Japan), and *Lotus japonicus* (Miyakojima MG-20)—*Rhizophagus irregularis* (DAOM197198; Premier Tech, Rivière-du-Loup, Canada) were used for observations of extraradical hyphae and mycorrhizal roots, respectively. Extraradical hyphae had to be harvested without contamination of soil particles that could make it impossible to section by a glass knife later. Therefore, onion seedlings raised from surface-sterilized seeds were inoculated with *G. margarita* onto a cellulose acetate membrane (47 mm in diam., 0.8 μm pore size; ADVANTEC, Tokyo, Japan) set in a root box system (Fig. 1A). The root box was made from a Petri dish, from which the side walls of the lid and bottom were partially removed (about 2 cm long). The bottom of the Petri dish was filled with autoclaved soil (river sand : field soil : horticultural soil = 5:4:1) and the surface of the soil was covered by a horticultural non-woven sheet. The cellulose acetate membrane was placed in the center of the sheet, an onion seedling was placed in the center of the membrane and 30 spores of *G. margarita* were inoculated close to the root. The inoculated plants were covered by the lid and kept in a growth room at 25°C (16 h light/8 h dark) in a vertical position with watering every day. From 10 days after the start of inoculation (dai), phosphate-reduced half strength macro-elements of Long-Ashton solution (2.5 mM NH_4_NO_3_, 25 μM NaH_2_PO_4_, 1 mM K_2_SO_4_, 2 mM CaCl_2_, 0.75 mM MgSO_4_, pH 5.8) were added to the plants twice a week. From 80 to 86 days, 50 ppm Cd nitrate in water was added every day until harvest. More than 10 onion seedlings were established and 4 plants were harvested and processed for further experiments.

Seedlings of *L. japonicus* raised from a surface-sterilized seed were transferred to soil (field soil : river sand = 2:1) and inoculated with 500 spores of *R. irregularis* close to the roots. In this system, two different Cd treatments were used. In one, an inoculated seedling was grown in the soil containing 200 ppm Cd (as CdCl_2_) in a pot (10.5 cm diam.) (1 compartment system, C1; Fig. 1B). In the other, in an acrylic box (14 cm length × 10 cm width × 10 cm depth), which was divided into five compartments along the length by nylon mesh sheets (37 μm) (5 compartments system, C5; Fig. 1C), a seedling was inoculated with the fungus in the center (root compartment). The two outermost compartments (hyphal compartments) were filled with soil (field soil : river sand = 3:1) containing 200 ppm Cd chloride. Buffer compartments were placed between the root and hyphal compartments, so that only extraradical hyphae were exposed to Cd. Each compartment system had two replicates. These pots were maintained in a growth chamber for 60 days.

### Sample preparation for SR-μXRF

*G. margarita* extraradical hyphae on the cellulose acetate membrane (Fig. 1D) were carefully collected using forceps and transferred to a formvar membrane formed around a copper loop with a short handle (3 mm diameter, 15 mm long) (12). The mycelium on the loop was further covered by a formvar membrane (3 mm × 6 mm) floating on water, and excess water on the loop was removed by a piece of filter paper and quickly plunged into liquid propane cooled with liquid N (VFZ-1; Vacuum Device Inc., Mito, Japan). Small pieces of mycorrhizal roots of *A. cepa* were longitudinally cut in half by a razor blade under a binocular microscope to confirm the colonization area. The colonized root pieces were transferred on copper loops, processed in the same way as the mycelium, and plunged into liquid propane. Small pieces of mycorrhizal roots of *L. japonicus* were cut in half, and the colonization area was confirmed and processed by a high-pressure freezing method. The small root pieces were placed in a holder filled with 1% agar and then high-pressure frozen by a Bal-Tec high pressure machine HPM 010 (BAL-TEC; Bal-Tec A.G., Balzers, Liechtenstein). All frozen materials were freeze substituted with dried acetone at −80°C containing Molecular Sieves 4A 1/16 (Wako, Osaka, Japan) for 3 days, and then the temperature was raised sequentially from −20°C to 4°C and then room temperature for 2 h each. Dried materials were then embedded in Spurr’s resin and polymerized at 60°C overnight. Resin-embedded materials were semi-thin sectioned using an ultramicrotome (Leica EM UC7, Vienna, Austria). A subsample of serial sections were stained with Toluidine blue O (TBO, 0.05% in 1% sodium borate in H_2_O) for observation of fungal structures under a light microscope (Axio Imager A1, Carl Zeiss, Jena, Germany) and other parts were stained with 4′, 6-diamidino-2-phenylindole (DAPI, 5 mM in H_2_O; Molecular Probes, Eugene, OR, USA) for observation of polyP within hyphae in fluorescent mode (UV emission, LP420 filter) under the microscope. From the resin block, a serial section (10 μm thick) was cut and mounted on a drop of water placed on a polypropylene (PP) membrane (6 μm thickness roll sheet; Rigaku Corporation, Tokyo, Japan) lying on a glass slide. The glass slide was heated on a hot plate to extend and fix the section on the PP membrane. After fungal structures in the sections were recorded using the differential interference mode of the light microscope, the PP membrane was cut and the edges were attached to an acrylic plate (40 mm × 40 mm, 1 mm thickness) having a hole (5 mm diam.) with adhesive acetate film tape (Scotch^®^ mending tape, Sumitomo 3M, Tokyo, Japan) over the section at the center of the hole. Images were captured with a digital CCD camera (DP70, Olympus, Tokyo, Japan).

### Cd and Zn mapping by SR-μXRF

**Fig. 2:**
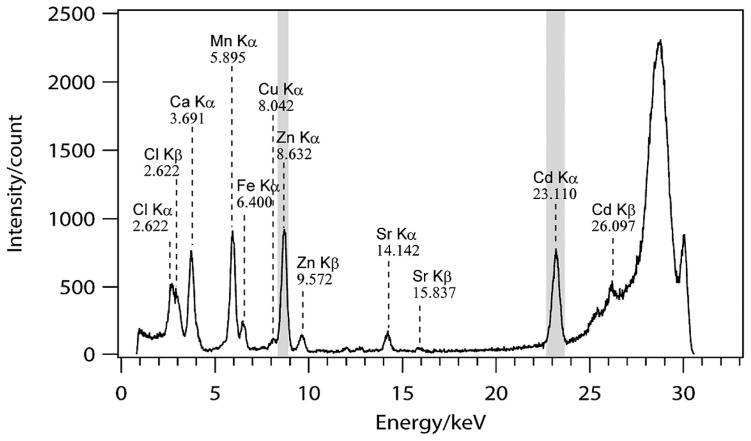
An X-ray fluorescence spectrum of an arbuscule of resin-embedded *Lotus japonicus*—*Rhizophagus irregularis* mycorrhizal root taken by a synchrotron irradiation microbeam (BL37XU; SPring-8). Shading shows energy ranges used for Zn and Cd mapping.

Elemental maps for Cd and Zn were obtained using the SR-μXRF system of BL37XU (SPring-8, Hyogo, Japan). Detailed machinery configurations and settings of the beam line are described elsewhere (9). Excitation X-ray energy was 30 keV, beam size was 1.3 μm × 0.86 μm and energy ranges (keV) for detection of Cd and Zn were 22.60–23.53 and 8.38–8.87, respectively (Fig. 2). First, rough maps were obtained with scanning conditions of 10 μm steps and 0.5 s exposure time to find the region where Cd was accumulated. Then the region of interest was focused by fine mapping with 1 or 0.5 μm steps and 0.5 or 1 s exposure time, respectively.

### P mapping by EDS-SEM and polyP observation by fluorescent microscopy

The thick sections analyzed by the SR-μXRF were excised by cutting out the PP membrane below so that it was larger than the sections, and attached to an aluminum holder with double-faced carbon tape for SEM. The specimen was carbon coated using a carbon coater (5 s × 2 times, JEOL JEC-530) and analyzed with a field emission SEM equipped with EDS (JSM-6340F; Oxford Link ISIS, Oxford instruments, Abingdon, UK) at accelerating voltage at 20 keV. Element mapping was conducted for P and the spectrum was measured in the area of interest. The sections on the holder used for EDS-SEM were then directly stained with DAPI, and the aluminum holder was place on a glass slide and subjected to observation under a fluorescent microscope at the same setting as for the semithin sections described above.

**Fig. 3:**
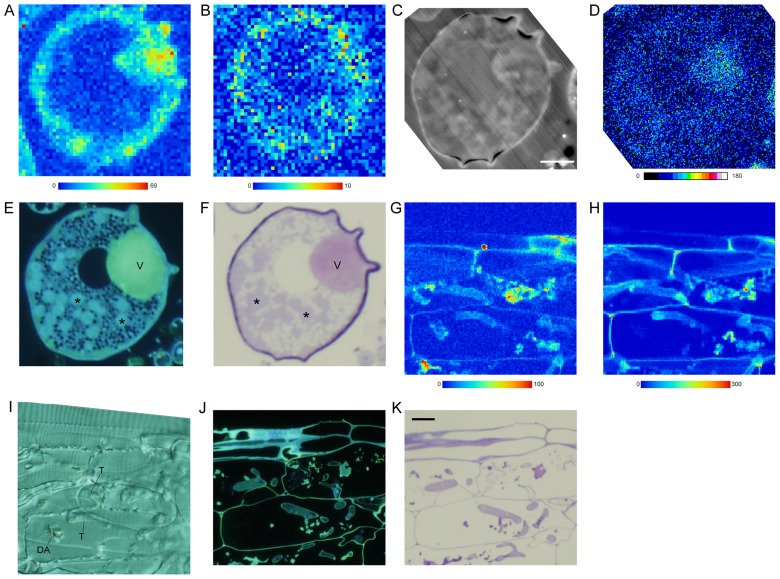
Plunge-frozen auxiliary cells of *Gigaspora margarita* produced on an extraradical hypha (A–F) and mycorrhizal roots (G–K) of *Allium cepa* mycorrhiza exposed to Cd solution. A, B, G, & H, Synchrotron radiation micro X-ray fluorescence (SR-μXRF) imaging of Cd (A, G), and Zn (B, H) (step, 1 μm; exposure time, 1 sec). Color bars indicate minimum and maximum X-ray counts. C & D. SEM-COMPO images of the section used for SR-μXRF (C) and phosphorus (Kα, 3.3 keV) mapping by EDS-SEM (D). Color bar indicates x-ray counts described by gray values. E & J, 4′,6-diamidino-2-phenylindole staining for polyphosphate of a serial semi-thin section observed by a fluorescence microscope. F & K, Toluidine blue O staining of a serial semi-thin section viewed by a light microscope. I, A differential interference image of a 10 μm thick section used for SR-μXRF. Asterisk, nucleus. DA, dead arbuscule; T, trunk hypha; V, vacuole. Bar: C, 10 μm; K, 20 μm.

Correlation between gray scales of Cd examined by SR-μXRF and P by EDS-SEM within a vacuole of an auxiliary cell of *G. margarita* was examined according to the following methods. Images of Cd (Fig. 3A) and P (Fig. 3D) were aligned using ImageJ software, and 30 circle areas (8 pixels in diameter) were arranged in a single layer on an entire vacuole (Fig. 6 right lower image). Average values of P and Cd within each circle (8 pixels in diameter) were obtained, and Pearson’s correlations coefficient between Cd and P gray values of 30 data sets were analyzed using Excel 2007.

### Ultrastructural observation by SEM

Ultrastructure of a resin-embedded *L. japonicus*—*R. irregularis* mycorrhiza, which was high pressure frozen and freeze substituted, was observed by a combined focused ion beam and scanning electron microscope (AURIGA 40 Crossbeam system; Carl Zeiss Microscopy GmbH, Oberkochen, Germany). All side walls besides the top surface of the resin block were covered by silver paint to avoid charging. After deposition of platinum to protect the region of interest, a view channel for SEM observation was milled into the block face using a 16 nA FIB probe current. The coarse cross-section was fine polished using a FIB milling (current of 2nA) and a SEM image was taken at an acceleration potential of 1.25 kV, aperture size of 60 μm, high current mode, and in-column energy selective backscattered (EsB) electron detection.

## Results

Plunge-frozen extraradical mycelia of *G. margarita* showed that Cd was localized in cell walls, cytoplasm, and vacuoles of auxiliary cells (Fig. 3A) and extraradical hyphae (data not shown). Among the cellular structures, vacuoles showed the highest signal, and the cytoplasmic region was weakest. In the cell, Zn signal was weak for detection of clear specific distribution (Fig. 3B). EDS-SEM elemental mapping of the same section showed that the vacuole accumulated P (Fig. 3C, D). DAPI staining (Fig. 3E) and TBO staining (Fig. 3F) of the cell confirmed that polyP was present in the vacuole as a dispersed form; polyP with the former stain emits characteristic yellow light under UV irradiation and with the latter it stains pink. In mycorrhizal roots of *A. cepa*, Cd (Fig. 3G) and Zn (Fig. 3H) were localized in host and fungal cell walls; however, the former element was localized in dead arbuscules but the latter was not (Fig. 3I). DAPI staining (Fig. 3J) and TBO staining (Fig. 3K) of the mycorrhizal roots showed that polyP was present in the vacuoles as a dispersed form in intraradical hyphae.

**Fig. 4:**
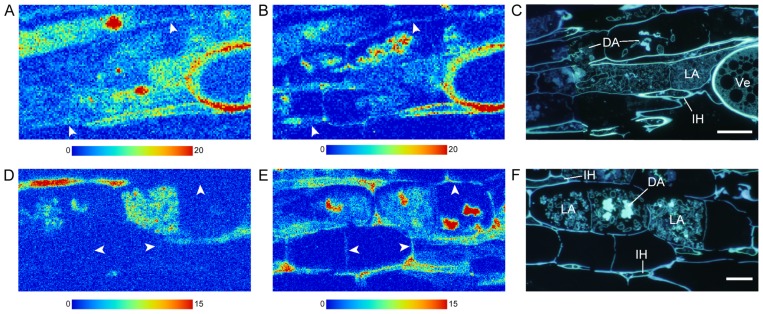
High-pressure-frozen *Lotus japonicus* and *Rhizophagus irregularis* mycorrhizal roots cultivated in the 1-compartment system (whole mycorrhizal root system was exposed to Cd; A–C; Bar: C, 20 μm) and in the 5-compartment system (only extraradical hyphae were exposed to Cd; D–F; Bar, F, 20 μm). A, B, D, & E, Synchrotron radiation micro X-ray fluorescence imaging of Cd (A, D), and Zn (B, E) (A & B: 1 μm step; 0.5 sec exposure time; D & E: 0.5 μm step; 0.5 sec exposure time). Color bars indicate minimum and maximum X-ray fluorescence counts. C & F, 4′,6-diamidino-2- phenylindole staining for polyphosphate of a serial semi-thin section observed by a fluorescence microscope. Arrowhead, host cell wall; DA, dead arbuscule; IH, intercellular hypha; LA, live arbuscule; Ve. Vesicle.

**Fig. 5:**
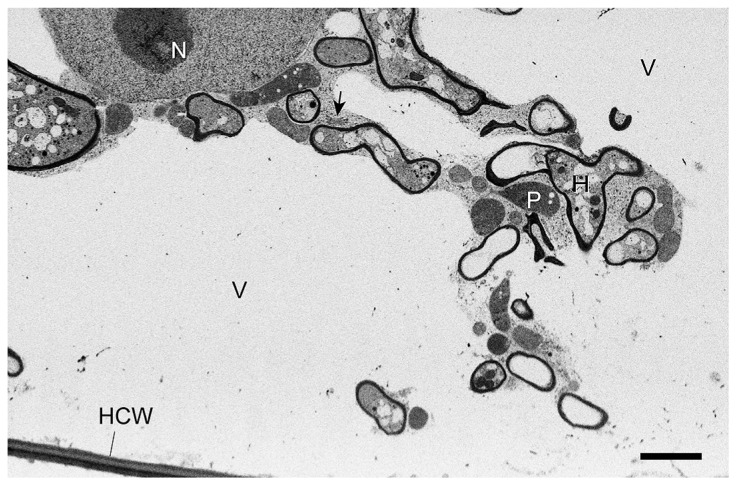
A combined focused ion beam and scanning electron micrograph of *Lotus japonicus* and *Rhizophagus irregularis* mycorrhiza which were grown in the C5 compartment system (only extraradical hyphae were exposed to Cd-contaminated soil) and high-pressure frozen and freeze substituted. Arrow, Golgi body, HCW, host cell wall; H, arbuscule fine hypha; N, nucleus; P, plastid; V, vacuole. Bar = 2 μm.

In high-pressure-frozen *L. japonicus* mycorrhizal roots (Fig. 4A–C), both Cd (Fig. 4A) and Zn (Fig. 4B) were localized in cell walls of the host, live and dead arbuscules, vesicles and intercellular hyphae when whole mycorrhizas were exposed to Cd by C1 treatment. In C5 treatment where only extraradical hyphae were exposed to Cd (Fig. 4D–F), the Cd signal was strong in live arbuscules and intraradical hyphae, and was not observed in host cell wall (Fig. 4D). Zn was localized in host cell walls and fungal structures where dead arbuscules showed the highest accumulation (Fig. 4E, F). Ultrastructure of high-pressure-frozen and freeze-substituted mycorrhizal roots showed superior preservation of both host and fungal structures and membranes including vacuoles without any sign of damage by water crystallization (Fig. 5).

**Fig. 6:**
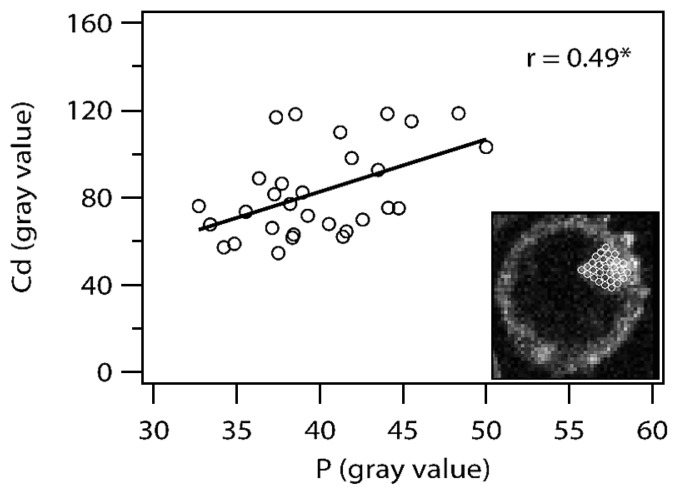
Correlation between gray scales of cadmium (Cd) examined by synchrotron radiation μ X-ray fluorescence and phosphorus (P) by energy dispersive X-ray fluorescence of SEM within a vacuole of an auxiliary cell of *Gigaspora margarita*. Thirty data sets of an average value within a circle (8 pixels in diam.) were obtained, where 30 circles were arranged in a single layer on an entire vacuole. Right lower image: an overlaid image of Cd distribution and an array of 30 circles on an entire vacuole. r, Pearson’s correlation coefficient; *, P < 0.01.

The correlation between Cd (Fig. 3A) and P (Fig. 3G) distribution in a vacuole of an auxiliary cell of *G. margarita* was examined, and gray values of Cd and P were statistically positively correlated (*P* <0.01, *r* = 0.49; Fig. 6).

## Discussion

This is the first study to apply high-energy synchrotron radiation (SR) to observe the localization of Cd at the cellular level in resin-embedded sections of mycorrhizal tissues, which were prepared by plunge- or high-pressure freezing techniques. Cd is not an essential element in organisms. Involvement of metal transporters in Cd uptake, such as the zinc transport family (ZIP family) and divalent metal transporter (DMT), have been reported in mammals (8), plants (21), and fungi (3). Proposed mechanisms for heavy metal tolerance or detoxification in organisms are binding with extracellular matrix (*e.g.* cell wall) or intracellular molecules (*e.g.* metallothioneins, phytochelatins, polyPs), sequestration in cellular compartments (*e.g.* vacuole), and active effluxes from the cell (5). In the mycorrhiza system, localizations of Zn, Cu, and Cd were investigated in extraradical mycelium and spores of monoxenically cultured *Glomus intraradices* DAOM197198 (the same strain used in this study, which is now *Rhizophagus irregularis* (19)), using EDS, where the heavy metals, Zn, Cu, and Cd, accumulated mainly in the fungal cell wall and in the vacuoles (7). Fungal vacuoles are tubular (2, 18) and store polyP together with potassium (16). The probability of the co-localization of potassium and Cd in vacuoles is problematic in EDS because the energy peak of characteristic XRF of Cd (Lβ1, 3.316 keV) overlaps that of potassium (K Kα, 3.312 keV). The use of high-energy SR X-ray allows detection of the Cd Kα line (23.11 keV), which eliminates the possibility of signal contamination from co-existing lighter elements; therefore, BL37XU of SPring-8 was selected as an analysis tool because of its characteristic micro beam line. One of the advantages of high-energy SR is that XRF analysis can be performed under ambient conditions, unlike techniques that use electrons as a primary beam, which generally require a high-vacuum condition for measurement. SR-μXRF has been applied to study the distribution of toxic metals and metalloids, such as Cd (9), and arsenic (11), in live plants. In mycorrhiza, fungi colonize between and inside the host cells, and also the host and fungal cells are compartmentalized. Therefore, dissection of the tissue is essential to analyze the cellular localization of elements. At the SR-μXRF of SPring-8 (BL37XU), freezedried plant sections were used to observe the distribution of metal elements on tissue scale (22, 23). Generally cryo-sectioning followed by freeze drying is considered a reliable technique to localize elements of biological tissues, most of which are in mobile forms. However, this technique has also disadvantages, such as the difficulty in obtaining good sections, and poor structural information, which are critical in endomycorrhizal tissues where colonization areas cannot be determined from outside of roots, and fungal live/dead statuses have to be considered for analyses. Therefore, in this study, the application of resin-embedded sections to SR-μXRF was examined as to whether it is reliable to observe the localization of Cd in mycorrhizal cells, because of the superiority of structural information over the cryo-sectioning method.

There are two major preparation categories to embed biological materials in resin. One is a conventional method of chemical fixation using aldehyde followed by ethanol dehydration, and the other is rapid freezing and freeze substitution. Ashford and co-workers reported that polyP granules observed in spherical vacuoles of hyphae were precipitated during the ethanol dehydration process in chemical fixation methods (16), and their results supported that fungal vacuoles are tubular where polyP is found in a dispersed form (2, 18). They also demonstrated that a high amount of Ca co-localized with polyP granules in chemically fixed materials was an artifact, and potassium was a counter ion of dispersed- form polyP in materials prepared by plunge freezing and freeze substitution (16). Tubular forms of vacuoles have been demonstrated in extraradical and intraradical hyphae of *G. margarita* using a live cell staining technique (20), and it was confirmed that the vacuoles of extraradical hyphae were filled with a dispersed form of polyP using plunge freezing and freeze substitution and resin- embedding techniques (12). In this study, plunge-frozen *A. cepa*—*G. margarita* mycorrhiza showed a dispersed form of polyP in vacuoles of both extraradical and intraradical hyphae, which indicated that this method was reliable for the study of sub-cellular structures of the fungus within roots. On the other hand, plant tissues are composed generally of large cells, and most of the cell volume is occupied by vacuoles. This characteristic makes it difficult to avoid ice crystal formation, which damages the structure. High-pressure freezing is the best technique to preserve plant tissues. Therefore, rapid-freeze techniques were used to localize Cd in mycorrhizal tissues to minimize structural and elemental re-location and replacement. This is the first study of an arbuscular mycorrhizal root system prepared by high-pressure freezing to show excellent preservation of both fungal and root membrane systems.

This study used two mycorrhizal systems (*A. cepa*—*G. margarita* and *L. japonicus*—*R. irregularis*), three different Cd treatments (addition of a Cd in solution during growth, and plants grown in Cd-contaminated soil [C1 & C5]), two mycorrhizal tissues (extraradical hyphae and mycorrhizal roots), and two freezing fixation techniques (plunge freezing and high-pressure freezing). When *A. cepa* and *L. japonicus* mycorrhizal roots were exposed to Cd, the element was localized in both host and fungal cell walls. However, C5 treatments where only extraradical hyphae of *L. japonicus* mycorrhiza were exposed to Cd showed a distinct difference in Cd distribution; the element was localized only in fungal structures in the roots. Because C1 and C5 materials presented in this study were prepared with the same fixation, dehydration, and embedding methods, the different localization patterns of Cd in host cells between these materials were caused by the Cd treatment difference of whether mycorrhiza roots or extraradical hyphae were exposed to Cd. The biological meaning of this difference will be discussed elsewhere. This Cd localization pattern of the C5 sample was also observed in resin sections of *L. japonicus* mycorrhizal roots prepared by a rapid freezing-freeze substitution method, and by a conventional chemical fixation method (2.5% glutaraldehyde–2% paraformaldehyde in 50 mM PIPES buffer followed by ethanol series dehydration) (data not shown). Furthermore, the higher signal of Cd in fungal cell walls and vacuoles, and lower in the cytoplasm, observed in plunge-frozen *G. margarita* mycelium, indicated that Cd was sequestrated within the fungal vacuoles, a structure bounded by a membrane, as a dispersed form. These differences in the localization of Cd within tissues and cells strongly suggested that Cd re-distribution during freeze-substitution and resin embedding did not occur or was negligible.

A marked advantage of the resin-embedding method over cryo-sectioning is the ability to combine information about structures and other different cellular information from the same and/or serial sections. Results of this study provided direct evidence of the co-localization of Cd and polyP in vacuoles of AM fungi. In *G. margarita* extraradical hyphae where Cd solution was added to the mycorrhiza, Cd was detected in the overall cell area; however, together with the highest signal in the cell wall, selective accumulation of vacuoles was evident and the cytoplasm signal was lowest. The vacuole was filled with polyP as the dispersed form, and a positive correlation of Cd and P signal was revealed. PolyP is a negatively charged macromolecule (13). Therefore, it is suggested that polyP plays a role as a counter ion of Cd in vacuoles and is involved in the translocation or detoxification of Cd within hyphae. This study revealed that SR-μXRF imaging combined with resin-embedded sections is powerful to determine Cd distribution in biological tissues at a subcellular level. Further studies are necessary to elucidate Cd transport, translocations, and transfer in AM symbioses.
